# Diagnostic accuracy systematic review of rectal bleeding in combination with other symptoms, signs and tests in relation to colorectal cancer

**DOI:** 10.1038/sj.bjc.6605426

**Published:** 2009-11-24

**Authors:** M Olde Bekkink, C McCowan, G A Falk, C Teljeur, F A Van de Laar, T Fahey

**Affiliations:** 1 Department of General Practice, Royal College of Surgeons in Ireland Medical School, Beaux Lane House, Lower Mercer Street, Dublin 2, Republic of Ireland; 2 Department of Primary and Community Care, Radboud University Nijmegen Medical Centre, P.O. 9101, 6500 HB, Nijmegen, The Netherlands; 3 Health Informatics Centre, Division of Clinical & Population Sciences & Education, University of Dundee, MacKenzie Building, Kirsty Semple Way, Dundee, DD2 4BF, Scotland; 4 Department of Public Health and Primary Care, Trinity College Dublin, Trinity Centre for Health Sciences, AMiNCH, Tallaght, Dublin 24, Ireland

**Keywords:** rectal bleeding, diagnosis, colorectal cancer, primary care

## Abstract

**Background:**

Rectal bleeding is a recognised early symptom of colorectal cancer. This study aimed to assess the diagnostic accuracy of symptoms, signs and diagnostic tests in patients with rectal bleeding in relation to risk of colorectal cancer in primary care.

**Methods:**

Diagnostic accuracy systematic review. Medline (1966 to May 2009), Embase (1988 to May 2009), British Nursing Index (1991 to May 2009) and PsychINFO (1970 to May 2009) were searched. We included cohort studies that assessed the diagnostic utility of rectal bleeding in combination with other symptoms, signs and diagnostic tests in primary care. An eight-point quality assessment tool was produced to assess the quality of included studies. Pooled positive likelihood ratios (PLRs), sensitivities and specificities were calculated.

**Results:**

Eight studies incorporating 2323 patients were included. Average weighted prior probability of colorectal cancer was 7.0% (range: 3.3–15.4%, median: 8.1%). Age ⩾60 years (pooled PLR: 2.79, 95% confidence interval (CI) 2.00–3.90), weight loss (pooled PLR: 1.89, 95% CI: 1.03–3.07) and change in bowel habit (pooled PLR: 1.92, 95% CI: 0.54–3.57) raise the probability of colorectal cancer into the range of referral to secondary care but do not conclusively ‘rule in’ the diagnosis. Presence of severe anaemia has the highest diagnostic value (pooled PLR: 3.67, 95% CI: 1.30–10.35), specificity 0.95 (95% CI: 0.93–0.96), but still only generates a post-test probability of 21.6%.

**Conclusions:**

In patients with rectal bleeding who present to their general practitioner, additional ‘red flag’ symptoms have modest diagnostic value. These findings have implications in relation to recommendations contained in clinical practice guidelines.

Patients presenting with symptoms of rectal bleeding commonly seek medical advice in primary care (Chaplin *et al*, 2000). A Dutch national survey on primary care revealed an incidence of rectal bleeding of 1.6 per 1000 (95% confidence interval (CI): 1.4–1.8) people in the general population seeking medical help from their general practitioner (Linden *et al*, 2004). However, the majority of patients with rectal bleeding in primary care do not have serious disease, with estimates of the risk of colorectal cancer varying between 2.4 and 11.0% (Douek *et al*, 1999; du Toit *et al*, 2006).

As rectal bleeding is a recognised early symptom of colorectal cancer, primary care has an important role in its early detection (Jones and Kennedy, 1999). Timely and efficient referral leading to early diagnosis of colorectal cancer may contribute to improved survival (Gondos *et al*, 2008). Current UK guidelines recommend urgent referral of patients aged 40 years and older who report rectal bleeding with a change of bowel habit towards looser stools and/or increased stool frequency persisting for 6 weeks or more. Patients aged above 60 years should be urgently referred if they have rectal bleeding alone or changed bowel habit without anal symptoms for 6 weeks or more (National Institute for Health and Clinical Excellence, 2005). Referring patients at low risk of colorectal cancer may lead to unnecessary harm (patient anxiety and iatrogenic harm from further diagnostic investigations) and longer waiting time for high-risk patients. An observational study in the United Kingdom reported an average time interval of 47 days between symptom presentation and diagnosis (Barrett *et al*, 2006).

The incidence of colorectal cancer in people experiencing rectal bleeding in the general population is <1 per 1000 people, and increases to 20–110 per 1000 patients in a primary care setting, and to 360 per 1000 patients in a secondary care setting (Fijten *et al*, 1994). These differing incidences reflect the increasing probability of colorectal cancer in each community and hospital setting. In general, two selection processes occur as a patient seeks medical help and is assessed in primary care. First, a patient with rectal bleeding decides whether or not to visit a general practitioner regarding his/her symptom of rectal bleeding. About 28–41% of people experiencing rectal bleeding consult their general practitioner (Crosland and Jones, 1995; Thompson *et al*, 2000). Second, their general practitioner performs a gate-keeping function, prioritising patients in terms of probability of colorectal cancer.

The aim of this diagnostic accuracy systematic review is to assess the additional diagnostic value of concurrent symptoms, signs and diagnostic tests in patients presenting to their general practitioner with rectal bleeding to stratify patients into low, medium or high probability groups of colorectal cancer and assist clinical decision making in primary care.

## Materials and methods

### Search strategy

An electronic search was performed using Medline (1966 to May 2009), Embase (1988 to May 2009), British Nursing Index (1991 to May 2009) and PsychINFO (1970 to May 2009). Combinations of MeSH terms and text words were used including: ‘Anal/ Rectal/ Colorectal/ Gastrointestinal’, ‘Bleeding/ Haemorrhage’, ‘Colorectal cancer/ Neoplasm’, ‘General Practice’, ‘Family Practice’ and ‘Primary Care’. Bibliographies and references of included studies, review articles and clinical guidelines were also searched. An unrestricted electronic search filter was used (Leeflang *et al*, 2008). No restrictions were placed on language.

### Study selection

Studies were independently selected by MOB and GF. If no consensus was achieved, studies were assessed by a third independent reviewer (TF). The inclusion and exclusion criteria were as follows:

- Population: unselected symptomatic patients recruited from a primary care setting presenting with the symptom of rectal bleeding.
- Study design: prospective cohort studies in a general practice setting. Other forms of observational studies, such as case–control studies were excluded. Screening studies and all types of retrospective studies were also excluded.
- Index test and reference standard: studies that investigate the diagnostic accuracy of symptoms, signs and diagnostic tests in relation colorectal cancer. Reference standard includes colonoscopy, flexible sigmoidoscopy, rigid sigmoidoscopy, barium enaema as well as follow-up over time.
- Outcome measures: Presence of colorectal cancer with data enabling the construction of 2 × 2 tables for the assessment of diagnostic accuracy of individual symptoms, signs or diagnostic tests.

### Quality assessment

An eight-point quality assessment tool was created to assess the quality of included studies. The assessment tool was applied by two independent reviewers (MOB, GF) and includes criteria from the studies by Whiting *et al* (2006) (QUADAS) and Laupacis *et al* (1997).

### Data extraction

Data from individual studies were independently extracted in duplicate by two reviewers (MOB, GF). If studies were eligible for inclusion, but data were insufficient to construct 2 × 2 tables, authors were contacted and asked to provide additional information.

### Statistical analysis

A weighted average prior probability was calculated by adding up the priors of the sub-studies, but multiplying the individual priors by the proportion of patients in the sub-study in relation to the total number of patients in all studies together, therefore, allowing larger studies to have more influence on the prior. It is calculated in a following way: ((prior study *X*_i_ × (*n*_studyXi_/*n*_total_)+…+prior study *X*_i+x_ × (*n*_studyXi+x_/*n*_total_))/number of included studies.

For the meta-analyses, a bivariate, random effects approach was used (Reitsma *et al*, 2005). The bivariate, random effects model focuses on estimating an average sensitivity and specificity, also estimating the unexplained variation in these parameters and the correlation between them. A summary estimate with a corresponding confidence bound of the average sensitivity and specificity across studies was computed for each symptom and sign. The bivariate, random-effects model along with the hierarchical summary receiver operating characteristic method are recommended over the more traditional methods of meta-analysis (Harbord *et al*, 2008). The DiagMeta package in R was used for the meta-analyses in which data from at least four studies were available (R Development Core Team, 2008), otherwise summary receiver operating characteristic curves were constructed using the random effects DerSimonian–Laird model (DerSimonian and Laird, 1986).

In terms of estimating the clinical value of symptoms, signs and test results, pooled likelihood ratios are estimated. Likelihood ratios are the most accessible way to refine clinical diagnosis on the basis of symptoms, signs and test results (Grimes and Schulz, 2005). A likelihood ratio >1 indicates an increase in probability of colorectal cancer, whereas a likelihood ratio <1 is associated with a decrease in the probability colorectal cancer.

## Results

**Figure 1:**
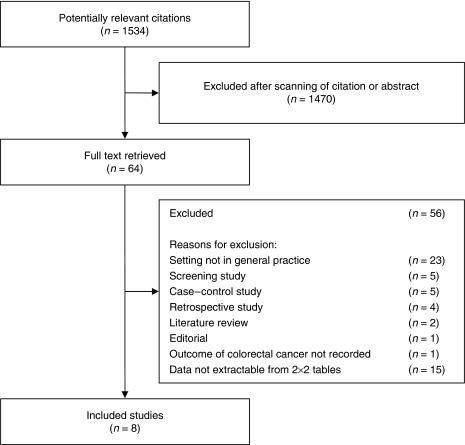
Flow of studies through review process.

The search strategy identified 1534 potential relevant citations. Eight studies met our inclusion criteria and were included in the final analysis (Figure 1; Mant *et al*, 1989; Fijten *et al*, 1995; Metcalf *et al*, 1996; Norrelund and Norrelund, 1996; Wauters *et al*, 2000; Ellis and Thompson, 2005; Heintze *et al*, 2005; du Toit *et al*, 2006).

### Characteristics of the included studies

The eight studies included 2323 patients and were carried out in primary care settings in England (Metcalf *et al*, 1996; Ellis and Thompson, 2005; du Toit *et al*, 2006), the Netherlands (Fijten *et al*, 1995), Germany (Heintze *et al*, 2005), Denmark (Norrelund and Norrelund, 1996), Belgium (Wauters *et al*, 2000) and Australia (Mant *et al*, 1989). The mean weighted prior probability of colorectal cancer was 7.0% (range: 3.3–15.4%, median: 8.1%). All studies included patients presenting with rectal bleeding in primary care and assessed the diagnostic accuracy of additional symptoms, signs and diagnostic tests. Summary characteristics of each included study are presented in Table 1.

### Quality of the included studies

**Figure 2:**
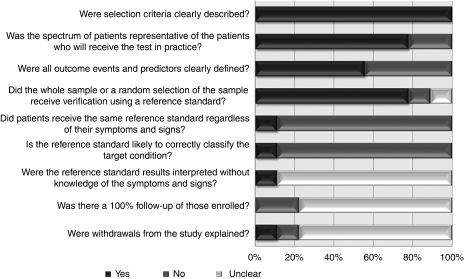
Summary diagram of the quality assessment of included studies.

The quality assessment of individual studies is presented in Table 2. In six studies, either the entire population or a random selection of the eligible population were subjected to a reference standard. The remaining two studies did not apply a reference standard to any of the included participants (Heintze *et al*, 2005; du Toit *et al*, 2006). Only one study applied the same reference standard (colonoscopy) to all included participants (Metcalf *et al*, 1996). Blinding of outcome assessment was poorly reported (Table 2). A summary diagram of the quality assessment is shown in Figure 2.

### Definition of the reference standard test and follow up

A variety of reference standards were used: colonoscopy, rigid sigmoidoscopy with (double contrast) barium enaema, air-contrast barium enaema, flexible sigmoidoscopy, flexible sigmoidoscopy and questionnaire, a questionnaire only, barium enaema only, and proctoscopy with sonography. Only two studies describe how many patients underwent a particular reference standard investigation (Mant *et al*, 1989; Metcalf *et al*, 1996). Follow-up was adequately described in three of the eight studies and ranged from, at least, 12 to 32 months (Fijten *et al*, 1995; Norrelund and Norrelund, 1996; Wauters *et al*, 2000). Follow-up was carried out by either sending recall letters to the general practitioner to obtain the number of all the new cases of cancer (Wauters *et al*, 2000), microscopic verification of colorectal cancer or an yearly letter to the general practitioner (Norrelund and Norrelund, 1996), or by checking medical records and information provided by the general practitioner (Fijten *et al*, 1995).

### Diagnostic value of rectal bleeding and related symptoms, signs and diagnostic tests

In the primary studies, all patients had rectal bleeding and presented with additional symptoms. The pooled positive likelihood ratios (PLRs), sensitivities and specificities for individual symptoms, signs and diagnostic tests are presented in Table 3. Overall, the magnitudes of the pooled PLRs are modest, with no individual symptom, sign or diagnostic test able to alter the probability of colorectal cancer into a definite range of ‘ruling in’ or ‘ruling out’ the diagnosis of colorectal cancer. Even classical symptoms, such as a history of weight loss and anaemia yield a modest pooled positive likelihood ratio of 1.89 (95% CI: 1.03–3.07) and 3.67 (95% CI: 1.30–10.35), respectively. Pooled sensitivities are low, varying from 0.17 to 0.62. Weight loss and anaemia yield a pooled specificity of 0.91 (95% CI: 0.83–0.96) and 0.95 (95% CI: 0.93–0.96), respectively (Table 3).

## Discussion

### Principal results

No individual symptom, sign or diagnostic test in patients with rectal bleeding is likely to shift the probability of colorectal cancer to the extent of ‘ruling in’ or ‘ruling out’ the diagnosis with any degree of certainty. Even the presence of anaemia (<12.0 g per 100 ml for women and <13.3 g per 100 ml for men) produces a shift in post-test probability to 21.6% (assuming a prior probability of 7.0%), a level that requires further diagnostic testing before colorectal cancer diagnosis is confirmed. ‘Red flag’ symptoms, such as weight loss and blood mixed with stool, seem to have only modest diagnostic value. Although the presence of these symptoms nearly doubles the post-test probability of colorectal cancer to about 13%, and their presence should ensure referral for further investigation, caution is needed when counselling patients about the possible reasons for their referral in terms of likely diagnoses. The fact that a presenting patient may be aged over 60 years also only provides modest diagnostic value in terms of probability of colorectal cancer (Table 3).

The findings from this systematic review have implications for clinical practice guidelines, showing that considerable diagnostic uncertainty is likely to exist in patients presenting to their general practitioner, even when they have additional symptoms, signs or test results that are conventionally associated with an increased risk of colorectal cancer (Hamilton and Sharp, 2004). The ideal threshold for referral is subject to several factors: individual patient's utilities or values regarding timely identification of colorectal cancer balanced against the iatrogenic harm and psychological damage of unnecessary investigation, and potential harm in patients free from disease. In addition, cost effectiveness of different referral thresholds in relation to probability of colorectal cancer also needs to be considered. To resolve these difficulties, formal cost utility estimates are required, which incorporate patient's utilities and cost at different referral thresholds.

### Context of previous studies

Our results differ from a recent UK case–control study that assessed the diagnostic value of clinical features of colorectal cancer before diagnosis. This study identified cases from a cancer registry and controls selected and matched in terms of age and registration with a general practice (Hamilton *et al*, 2005). In this case–control study, PLRs were considerably higher than found in this systematic review of cohort studies. For example, PLRs for weight loss (5.1), abdominal pain (4.5) and anaemia <10 g per 100 ml (9.5) would all be associated with definitive shifts in the probability of colorectal cancer (Grimes and Schulz, 2005). The most likely explanation for this discordant finding is that recall bias amongst controls may have produced a comparison group that did not remember having colorectal symptoms in the past, thus inflating estimates of diagnostic utility for symptoms, signs and diagnostic tests when compared with individuals with colorectal cancer (Grimes and Schulz, 2002).

Our results are more consistent with a recent diagnostic accuracy review of cohort studies that included patients in both primary and secondary care presenting with ‘alarm’ features (Ford *et al*, 2008). In their systematic review the overall conclusion was that most alarm features of colorectal cancer had poor sensitivity and specificity for the diagnosis of colorectal cancer. The presence of rectal bleeding (PLR: 1.32, 95% CI: 1.19–1.47), weight loss (PLR: 1.96, 95% CI: 1.25–3.08) or iron deficiency anaemia (PLR: 1.43, 95% CI: 0.75–2.74) do raise the probability of colorectal cancer but only to a modest extent. The results from this systematic review of cohort studies in primary care, in which rectal bleeding was an inclusion criterion, are broadly similar in relation to diagnostic utility of symptoms, signs and diagnostic tests.

Our findings suggest that older age and iron deficiency anaemia are predictive of colorectal cancer. These findings are consistent with several previous studies. Panzuto *et al* (2003) showed that age >50 years and iron deficiency anaemia are independently associated with colorectal cancer in primary care (odds ratios: 9.0 and 8.8 on multivariable analysis, respectively). Patients with right-sided bowel cancers have a significantly lower haemoglobin level at presentation than those with left-sided cancers (Yates *et al*, 2004; Masson *et al*, 2007). There seems to be a trade-off in relation to the diagnostic and prognostic value of the presence of anaemia and colorectal cancer, whereas presence of anaemia is most useful in ruling in a diagnosis of colorectal cancer, it is also associated with a more advanced disease and a poorer prognosis (Stapley *et al*, 2006).

Lastly, having a positive family history for colorectal cancer has been cited as being associated with an increased risk of current colorectal cancer (Bonelli *et al*, 1988; Slattery *et al*, 2003). The three included studies assessing family history of colorectal cancer yield varying and inconsistent likelihood ratios (Mant *et al*, 1989; Fijten *et al*, 1995; Heintze *et al*, 2005). Heintze *et al* (2005) calculated a PLR of 3.65, whereas Fijten *et al* (1995) and Mant *et al*, 1989) reported a PLR <1. More research is needed regarding the definition of positive family history, how it might relate to risk of colorectal cancer and the impact of using family history as a preliminary screening question prior to Faecal Occult Blood (FOB) screening programs (Polmear and Glasziou, 2008).

### Limitations of the present study

The validity of the results of this systematic review is determined by an independent, unbiased selection process. However, any systematic review may be susceptible to publication bias (Irwig *et al*, 1994, 1995; Deeks, 2001). The quality of the review is dependent on the quality of the included cohort studies. Several dimensions that relate to the quality of the included studies are unclear or inadequately reported (Table 2, online). This finding is not intended as a criticism of the original studies, but is more a reflection on the considerable challenges of undertaking cohort studies in primary care settings that rely on complete identification and follow-up of all eligible incident cases of rectal bleeding. For instance, in one included study, general practitioners were asked to include a maximum of three to four patients (Norrelund and Norrelund, 1996). This prior selection may lead to a preferential selection of more severe cases and subsequent spectrum bias, producing spurious clinical associations and overestimation of likelihood ratios (Jelinek, 2008).

The other feature of included studies was the application of a variety of different reference standards. In the detection of colorectal cancer, the most sensitive and specific diagnostic test is colonoscopy, followed by a flexible sigmoidoscopy in combination with a barium enaema (Irvine *et al*, 1988; Rex *et al*, 1990; Helfand *et al*, 1997). In the included studies, a variety of reference standard tests were used with a possibility of work-up bias in some studies as lower-risk patents were subject to less rigorous reference standard tests (Table 2). Other methodological problems include incomplete or inadequate blinding of outcome assessment and incomplete reporting on losses to follow-up (Table 2).

We found significant between-study heterogeneity for a range of symptoms and signs (Table 3). This might be due to whether or not rectal bleeding was the principal reason for consultation and also the duration of rectal bleeding. Three included studies report rectal bleeding as the primary complaint in 15%, 51% and 100% of patients, respectively, (Fijten *et al*, 1995; Metcalf *et al*, 1996; Wauters *et al*, 2000), and in terms of duration two studies excluded patients with rectal bleeding longer than six and twelve months, respectively (Mant *et al*, 1989; Metcalf *et al*, 1996).

For age categories, we calculated sensitivities, specificities and likelihood ratios. However, primary studies use different age cut-off points, which complicate the generation of reference categories. In our review, there is a slight overlap in some of the age categories, which may have affected the precision of pooled estimates. In terms of dark red blood, the reference category includes both patients with bright red blood and a colour in between. For a history of rectal bleeding the reference category consists of patients having a first episode of rectal bleeding.

### Future studies

There are considerable challenges with undertaking cohort studies of rectal bleeding in primary care, including recruitment of consecutive patients; eliciting full history of symptoms, signs and diagnostic tests; consenting ‘low risk’ patients to potentially unpleasant and invasive reference standard tests, and ensuring adequate follow-up of patients. Furthermore, the relative rarity of colorectal cancer in primary care makes it difficult to design a study large enough to yield adequate power to detected significant clinical predictors of colorectal cancer (Hamilton and Sharp, 2004).

Despite these challenges, this systematic review shows that the evidence base is not substantial at present and that further studies are required, which assess the diagnostic accuracy of lower gastrointestinal symptoms in community settings. A study assessing the combined value of rectal bleeding and additional symptoms has been undertaken in secondary care, showing that patients presenting with rectal bleeding and a change in bowel habit *without* perianal symptoms are at highest risk of colorectal cancer (PLR: 4.2). Patients with rectal bleeding and perianal symptoms, but *without* a change in bowel habit had lowest risk of colorectal cancer (negative likelihood ratio: 1.3) (Thompson *et al*, 2007). Similar studies, focussing on patients presenting in primary care are needed.

Future studies should recruit consecutive patients, apply an agreed reference standard to all patients and evaluate the combined value of symptoms, signs and diagnostic tests in the form of a clinical prediction rule (McGinn *et al*, 2008). Future primary studies should also report their data completely, preferably using the Standards for Reporting of Diagnostic Accuracy (STARD) criteria as guidelines (Bossuyt *et al*, 2003).

In conclusion, this systematic review shows that no individual symptom, sign or diagnostic test in patients with rectal bleeding is likely to conclusively raise the probability of colorectal cancer in primary care settings. Even conventionally stated ‘red flag’ symptoms, such as weight loss and blood mixed with stool, have modest diagnostic value. Future studies are needed to establish the diagnostic value of individual and combined symptoms, signs and diagnostic tests so that the evidence base for appropriate and timely referral is more secure.

## Figures and Tables

**Table 1:** Summary of the included studies

**Author, Year**	**Number of patients** **Mean age (range)** **Sex (♂:♀)**	**Patient population** **Setting**	**Prior colorectal cancer**	**Reference standard and number or percentage of patients receiving it** **Follow-up**	**Prevalence of symptoms/signs/patient characteristics**		**PLR**
Du Toit *et al*, 2006	265 pt ND years (45–ND years)♂ND: ♀ND	Pt ⩾ 45 years with new onset rectal bleeding, irrespective of other symptoms. Rural practice in England; four doctors; one registrar.	5.7% (15 of 265)	Rigid sigmoidoscopy with barium enaema (most patients), flexible sigmoidoscopy, or colonoscopy. (p 69) Follow-up: unclear	*Patient characteristics* Age 45–54 years Age 55–64 years Age 65–74 years Age ⩾75 years	% *Population* 19% 28% 24% 29%	*PLR* 0.7 0.2 1.8 1.4
							
Ellis and Thompson 2005	319 pt 59 years (35–94 years) ♂143: ♀176	Pt 35 years consulting their GP with rectal bleeding 19 GPs in 3 practices in the United Kingdom: 1 market town/rural community; 1 suburban; 1 inner-city	3.4% (11 of 319)	-Flexible sigmoidoscopy (219 pt) -Patient questionnaire (47 pt) -Flexible sigmoidoscopy & questionnaire (53 pt) -Barium enaema (37 pt) -Colonoscopy (24 pt) Follow-up:18 months	*Symptoms/signs/patient characteristics* Bleeding and CIBH (*n*=119) Bleeding and CIBH (loose +/− frequent) (*n*=83)	% *Population* 37% 26%	*PLR* 2.4 1.3
					Bleeding and no perianal symptoms (*n*=63)	20%	2.9
					Bleeding CIBH and abdominal pain (*n*=67)	21%	1.0
					Dark blood (*n*=31)	10%	2.1
					Age ⩾ 60 years (*n*=155)	49%	1.5
					Blood on paper only (*n*=82)	26%	0.6
					Large volume of blood (*n*=79)	25%	0.3
					First time rectal bleeding (*n*=106)	33%	1.2
					Blood mixed with stool (*n*=33)	10%	0.7
							
Fijten *et al*, 1995	269 pt 42 years (18–75 years) ♂118: ♀151	Patients ⩾ 18 years and ⩽75 years with overt rectal bleeding as a reason for consult or history of recent (<3 month) blood loss visible. 83 GPs in the South of the Netherlands	3.3% (9 of 269)	A total of 31% had further investigations initiated by the GP by means of sigmoidoscopy (9%) colon roentenography (9%), proctoscopy (8%), sonography (6%) and colonoscopy (2%). Some patients underwent more than one investigation. Follow-up: at least 1 year (mean 20 months) Medical records and information of the GP.	*Symptoms/signs* Blood seen: Mixed with stool only On stool or mixed with stool only Others or combinations	% *Population* 5% 20% 45%	*PLR* 8.0 3.8 0.4
					Abdominal pain	50%	0.7
					Change in bowel habit (more frequently or diarrhoea or variously, but not constipation)	29%	2.9
					Pain at night	19%	0.0
					Decreased appetite	16%	0.7
					Nausea	25%	0.4
					Weight loss	16%	3.0
					Family history of abdominal disease	31%	0.0
					Previous history of rectal bleeding	36%	0.0
					Pale conjunctivae	2%	5.8
					Perianal eczema	6%	6.2
					Rectal palpation (*n*=208):		
					Haemorrhoid	7%	2.5
					Tumour	0%	1.0
					Abnormal prostate	1%	22.3
					*Patient characteristics*	% *Population*	*PLR*
					Age 18–29 years	23%	0.0
					Age 30–39 years	26%	0.0
					Age 40–49 years	20%	0.0
					Age 50–59 years	15%	0.7
					Age 60–75 years	15%	7.2
					Male	44%	1.8
					*Laboratory test results*	% *Population*	*PLR*
					Anaemia (Hb♀ < 7.5 mmol l^−1^ ♂<8.5 mmol l^−1^)	5%	6.6
					ESR high (♀>28 mm h^−1^♂>8.5 mm h^−1^)	9%	4.2
					ESR high (>30 mm h^−1^)	4%	8.8
					High white blood cell count (>109 per litre) (*n*=219)	9%	5.8
					Haemoccult ⩾1 positive out of 3	15%	2.3
							
Heintze *et al*, 2005	422 pt ND years (ND–ND years) ♂199: ♀222	Patients >15 years 94 GPs in Germany	4.0% (17 of 422)	Diagnostic work-up: Sonography (52 pt) Rectoscopy (29 pt) Sigmoidscopy (26 pt) Colonoscopy (195 pt) Treatment by GP (93pt) Follow-up: Unclear	*Symptoms/signs/patient characteristics* Male Age <50 years Age ⩾50 years Age 15–24 years	% *Population* 53% 38% 62% 2%	*PLR* 1.3 0.2 1.5 0.0
					Age 25–34 years	11%	0.4
					Age 35–44 years	14%	0.3
					Age 45–54 years	16%	0.5
					Age 55–64 years	28%	1.3
					Age 65–74 years	18%	1.7
					Age 75–84 years	8%	0.5
					Age 85–94 years	2%	8.4
					Weight loss	3%	1.3
					Changed bowel habit	18%	1.2
					Abdominal pain	24%	0.7
					Anaemia	6%	2.4
					Dark red blood	12%	1.1
					Blood mixed with stool	19%	1.9
					Family history of colon carcinoma	7%	3.6
							
Mant *et al*, 1989	145 pt 58 years (40–95 years) ♂77: ♀68	Pt ⩾ 40 years who consulted the GP for rectal bleeding 48 GPs in Australia	11% (16 of 145)	-Total colonoscopy (104 pt) -Endoscopy to at least 30 cm and an air-contrast barium enaema (32 pt)-Investigations not complete, but an obvious source was found, e.g. rectal cancer at proctoscopy. (9 pt) Follow-up: unclear	*Symptoms/signs/patient characteristics* Male First-degree relative with CRC (*n*=143) Abdominal pain (*n*=144)	% *Population* 53% 14% 30%	*PLR* 0.8 0.9 0.8
					Change in bowel habit (*n*=143)	39%	1.0
					Feeling of incomplete evacuation of rectum	29%	1.1
					Weight Loss (*n*=143)	10%	1.3
					Anal itch	25%	0.2
					Pain on defecation	21%	0.6
					Anal protrusion noticed by patient	21%	0.3
					Dark red blood (*n*=144)	16%	1.7
					Blood mixed with faeces (*n*=140)	36%	2.2
					Haemorrhoids identified by GP	51%	0.5
Metcalf *et al*, 1996	99 pt 58 years (40–86 years) ♂42: ♀57	Patients ⩾ 40 years 17 GPs in Newcastle upon Tyne, England	8.1% (8 of 99)	Questionnaire (99pt) Colonoscopy (98pt) Barium enaema in any patients whom a satisfactory colonoscopy was not completed. (1pt) Follow-up: Unclear (Practices participated between 1–9 months)	*Symptoms/signs/patient characteristics* Dark red blood loss Blood mixed with stool Diarrhoea Associated slime	% *Population* 31% 46% 32% 28%	*PLR* 1.2 1.4 0.9 1.4
					Constipation	39%	0.3
					Change in bowel habit	39%	1.3
					Abdominal pain	42%	0.9
					Weight loss	15%	1.8
							
Norrelund and Norrelund 1996(1)	208 pt 42 years (18–75 years) ♂97: ♀111	Patients ⩾ 40 years presenting with a first episode of rectal bleeding 96 GPs from Denmark	15.4% (32 of 208)	GPs were asked to arrange either a barium enaema or a colonoscopy at the first consultation. Follow-up: 32 months Colorectal cancer microscopically verified or yearly letter to GP	*Symptoms/signs/patient characteristics* Male Age 40–69 years Age 70–79 years Age 80+ years	% *Population* 47% 68% 25% 7%	*PLR* 1.3 0.3 3.3 2.2
					Weight loss	11%	1.6
					Abdominal pain	23%	1.5
					Change in bowel habits	29%	2.6
					Discomfort	27%	1.3
							
Norrelund and Norrelund 1996(2)	156 pt 42 years (18–75 years) ♂71: ♀85	Patients ⩾ 40 years first bleeding episode or change in usual bleeding pattern 112 GPs from Denmark	14.1% (22 of 156)	GPs were asked to arrange either a barium enaema or a colonoscopy at the first consultation. Follow-up: 22 months CRC microscopically verified or yearly letter to GP	*Symptoms/signs/patient characteristics* Male Age 40–69 years Age 70–79 years Age 80+ years	% *Population* 46% 72% 21% 7%	*PLR* 1.0 0.7 2.4 0.6
					Weight loss	14%	1.8
					Abdominal pain	27%	2.2
					Change in bowel habits	31%	1.6
					Discomfort	26%	0.9
					New rectal bleeding	69%	0.8
							
Wauters *et al*, 2000	386 pt ND years (ND-ND years) ♂ND: ♀ND	Network of sentinel practices in Belgium	7.0% (27 of 386)	Investigations such as endoscopy were not systematically performed. ‘To obtain the number of all new cases of cancer, we sent recall letters to the practices every six months and at the end of the follow-up period.’ (p 998) Follow-up (clinical): 18–30 months	*Symptoms/signs/patient characteristics* Age <50 years Age 50–59 years Age 60–69 years Age 70–79 years Age ⩾ 80 years Pain	% *Population* 37% 15% 18% 17% 13% 9%	*PLR* 0.1 0.2 1.7 3.6 0.8 0.0
					Spasms	29%	0.8
					Weight loss	6%	2.5
					Palpable tumour	5%	6.1

Abbreviations: CIBH=change in bowel habit; ND=no data available. The page numbers refer to the original text of the included studies.

**Table 2:** Methodological standards for quality assessment of included studies

Study ID	Were selection criteria clearly described? (Whiting *et al*, 2006)	Was the spectrum of patients representative of the patients who will receive the test in practice? (Whiting *et al*, 2006)	Were all outcome events and predictors clearly defined? (Laupacis *et al*, 1997)	Did the whole sample or a random selection of the sample receive verification using a reference standard? (Whiting *et al*, 2006)	Did patients receive the same reference standard regardless of their symptoms and signs? (Whiting *et al*, 2006)	Is the reference standard likely to correctly classify the target condition? (Whiting *et al*, 2006)	Were the reference standard results interpreted without knowledge of the symptoms and signs? (Whiting *et al*, 2006)	Was there a 100% follow-up of those enrolled? (Laupacis *et al*, 1997) Were withdrawals from the study explained? (Whiting *et al*, 2006)
Du Toit *et al* (2006)	Yes	Yes	No	Unclear “A small number of patients may not have entered the diagnostic protocol, despite of frequent reminders.” (p 69)	No -Rigid sigmoidoscopy -Flexible sigmoidoscopy -Colonoscopy	Suboptimal Suboptimal Best method available	Unclear	-No -Withdrawals not explained
Ellis and Thompson (2005)	Yes	Yes	No	Yes	No -Flexible sigmoidoscopy (219 pt) -Patient questionnaire (47 pt) -Flexible sigmoidscopy & questionnaire (53 pt) -Barium enaema (37 pt) -Colonoscopy (24 pt)	Suboptimal Not suitable Suboptimal Suboptimal Best method available	Unclear	Unclear
Fijten *et al* (1994)	Yes	Yes	Yes	Yes	No -Sigmoidoscopy (8 pt) -Colon-roentenography (8 pt) -Proctoscopy (7 pt) Sonography (5 pt) -Colonoscopy (2 pt)	Suboptimal Suboptimal Suboptimal Suboptimal Best method available	Yes	-No -Yes “21 patients excluded because lost of follow-up. (moved to an unknown destination)”
Heintze *et al* (2005)	Yes	Yes	Yes	No The selection of patients having further investigation was not at random	No -Sonography (52 pt) -Rectoscopy (29 pt) -Sigmoidoscopy (26 pt) -Colonoscopy (195 pt)	Suboptimal Suboptimal Suboptimal Best method available	Unclear	Unclear
Mant *et al* (1989)	Yes	Yes	No	Yes	No -Total colonoscopy (104 pt) -Endoscopy to at least 30 cm and an air-contrast barium enaema (32 pt) - Investigations not complete, but an obvious source was found, for example, rectal cancer at proctoscopy. (9 pt)	Best method available Suboptimal Suboptimal	Unclear	Unclear
Metcalf *et al* (1996)	Yes	Yes	Yes	Yes	Yes Colonoscopy 98 pt Barium enaema 1 pt (because colonoscopy was impossible)	Best method available	Unclear “The questionnaire was re-administered by the colonoscopist before the procedure” (p162)	Unclear
Norrelund and Norrelund (1996) (1)	Yes	No Selection made during recruitment. GPs were allowed to include a maximum of 3 patients.	Yes	Yes	No GPs were asked to arrange either a barium enaema or a colonoscopy at the first consultation. (p161)	Suboptimal “Although the authors asked the GP to refer all patients for a full colon examination, but this was no inclusion criterion.” (p165)	Unclear	Unclear
Norrelund and Norrelund (1996) (2)	Yes	No Selection made during recruitment. GPs were allowed to include a maximum of 4 patients.	Yes	Yes	No GPs were asked to arrange either a barium enaema or a colonoscopy at the first consultation. (p161)	Suboptimal	Unclear	Unclear
Wauters *et al* (2000)	Yes	Yes	No Predictors clearly defined, but outcomes only colorectal cancer reported.	Yes	No “Our reference standard was colorectal cancer diagnosed during a clinical follow-up of 18–30 months. Investigations, such as endoscopy, were not systematically performed.” (p 998)	Suboptimal	Unclear	Unclear

Abbreviations: GP=general practitioners; pt=patient. The page numbers refer to the original text of the included studies.

**Table 3:** Clinical value of symptoms and signs in patients presenting with rectal bleeding in terms of colorectal cancer

	**No of studies^a^**	**No of patients**	**Sens**	**(95% CI)**	**Spec**	**(95% CI)**	**Pooled PLR**	**(95% CI)**
*Patient characteristics*								
Male	5	1253	0.58	(0.48–0.67)	0.52	(0.48–0.56)	1.21	(1.00–1.46)
Age <40 years^b^	2	745	0.03	(0.00–0.16)	0.73	(0.69–0.76)	0.32	(0.05–2.21)
Age 40–59 years^b^	4	1387	0.09	(0.04–0.19)	0.79	(0.70–0.86)	0.41	(0.18–0.90)
Age ⩾ 60 years^b^	6	1760	0.66	(0.45–0.83)	0.76	(0.68–0.83)	2.79	(2.00–3.90)
Family history colorectal cancer	3	886	0.15	(0.06–0.28)	0.85	(0.82–0.87)	1.05	(0.16–6.88)
								
*Symptoms*								
Dark red blood^c^	4	949	0.22	(0.13–0.34)	0.84	(0.69–0.93)	1.37	(0.59–3.30)
Weight loss	7	1737	0.17	(0.06–0.37)	0.91	(0.83–0.96)	1.89	(1.03–3.07)
Abdominal pain	7	1739	0.25	(0.04–0.62)	0.73	(0.52–0.89)	0.94	(0.19–1.59)
Changed bowel habit	5	1254	0.62	(0.18–0.94)	0.68	(0.53–0.80)	1.92	(0.54–3.57)
Blood mixed with the stool	5	1225	0.40	(0.04–0.93)	0.81	(0.23–0.98)	1.91	(0.75–5.51)
Previous history of rectal bleeding^d^	2	425	0.30	(0.05–0.41)	0.66	(0.63–0.71)	0.58	(0.14–1.41)
Perianal symptoms – pain on defecation	2	411	0.22	(0.13–0.36)	0.41	(0.22–0.78)	0.49	(0.25–0.97)
Perianal symptoms – itch/eczema	2	414	0.17	(0.07–0.33)	0.87	(0.73–0.95)	1.31	(0.25–6.21)
								
*Signs and diagnostic tests*								
Rectal palpation – haemorrhoid	2	354	0.24	(0.09–0.45)	0.73	(0.46–0.91)	0.51	(0.09–2.97)
Anaemia (Hb ♀<12.0 g per 100 ml ♂<13.3 g per 100 ml)	2	700	0.17	(0.05–0.35)	0.95	(0.93–0.96)	3.67	(1.30–10.35)

Abbreviations: CI=confidence interval; Hb, haemoglobin; PLR=positive likelihood ratio.

a Norrelund and Norrelund (1996) consists of two independent sub-studies, and therefore are independently assessed. In the column ‘no of studies’ these two substudies are counted as two separate studies.

b There is a slight age overlap between the individual studies.

c The reference category of dark red blood consists of patients having bright red blood or a colour in between.

d The reference category of previous history of rectal bleeding consists of patients having a first episode of rectal bleeding.
